# Multiparametric ultrasound: evaluation of greyscale, shear wave elastography and contrast-enhanced ultrasound for prostate cancer detection and localization in correlation to radical prostatectomy specimens

**DOI:** 10.1186/s12894-018-0409-5

**Published:** 2018-11-08

**Authors:** Christophe K. Mannaerts, Rogier R. Wildeboer, Arnoud W. Postema, Johanna Hagemann, Lars Budäus, Derya Tilki, Massimo Mischi, Hessel Wijkstra, Georg Salomon

**Affiliations:** 1 Department of Urology, Amsterdam University Medical Centers, University of Amsterdam, Amsterdam, The Netherlands; 2 Department of Electrical Engineering, Eindhoven University of Technology, Eindhoven, The Netherlands; 3 Martini Clinic, Prostate Cancer Center, University Hospital Hamburg-Eppendorf, Hamburg, Germany

**Keywords:** Prostate cancer, Imaging, Ultrasound, Multiparametric, Radical prostatectomy, Detection, Accuracy

## Abstract

**Background:**

The diagnostic pathway for prostate cancer (PCa) is advancing towards an imaging-driven approach. Multiparametric magnetic resonance imaging, although increasingly used, has not shown sufficient accuracy to replace biopsy for now. The introduction of new ultrasound (US) modalities, such as quantitative contrast-enhanced US (CEUS) and shear wave elastography (SWE), shows promise but is not evidenced by sufficient high quality studies, especially for the combination of different US modalities. The primary objective of this study is to determine the individual and complementary diagnostic performance of greyscale US (GS), SWE, CEUS and their combination, multiparametric ultrasound (mpUS), for the detection and localization of PCa by comparison with corresponding histopathology.

**Methods/design:**

In this prospective clinical trial, US imaging consisting of GS, SWE and CEUS with quantitative mapping on 3 prostate imaging planes (base, mid and apex) will be performed in 50 patients with biopsy-proven PCa before planned radical prostatectomy using a clinical ultrasound scanner. All US imaging will be evaluated by US readers, scoring the four quadrants of each imaging plane for the likelihood of significant PCa based on a 1 to 5 Likert Scale. Following resection, PCa tumour foci will be identified, graded and attributed to the imaging-derived quadrants in each prostate plane for all prostatectomy specimens. Primary outcome measure will be the sensitivity, specificity, negative predictive value and positive predictive value of each US modality and mpUS to detect and localize significant PCa evaluated for different Likert Scale thresholds using receiver operating characteristics curve analyses.

**Discussion:**

In the evaluation of new PCa imaging modalities, a structured comparison with gold standard radical prostatectomy specimens is essential as first step. This trial is the first to combine the most promising ultrasound modalities into mpUS. It complies with the IDEAL stage 2b recommendations and will be an important step towards the evaluation of mpUS as a possible option for accurate detection and localization of PCa.

**Trial registration:**

The study protocol for multiparametric ultrasound was prospectively registered on Clinicaltrials.gov on 14 March 2017 with the registry name ‘Multiparametric Ultrasound-Study for the Detection of Prostate Cancer’ and trial registration number NCT03091231

## Background

To date, patients with a clinical suspicion of prostate cancer (PCa) based on elevated serum prostate specific antigen (PSA) and/or a suspicious digital rectal examination (DRE) should undergo a transrectal ultrasound (TRUS)-guided systematic biopsy as next step in assessing presence of PCa [1]. This combination of tests results in a considerable rate of benign biopsy results, overdiagnosis of clinically insignificant PCa and underdiagnosis and undergrading of clinically significant PCa [2, 3]. Moreover, systematic transrectal biopsy carries significant morbidity [4]. As a consequence, the diagnostic pathway for PCa has begun to lean towards an imaging-driven targeted biopsy approach. Multiparametric magnetic resonance imaging (mpMRI) of the prostate and targeted biopsies of suspicious mpMRI lesions has evolved into an increasingly appealing tool in the PCa diagnostic arsenal and is currently recommended in men with a sustained suspicion of PCa after a negative initial biopsy [1]. The exact role for mpMRI in PCa diagnosis remains unclear, however; improved clinically significant PCa detection compared with systematic biopsy is controversial in biopsy-naïve patients and mpMRI as a triage test before biopsy seems to miss significant PCa [5, 6]. Moreover, universal implementation of an mpMRI pathway seems unlikely for now, given the relatively high cost, low specificity with high rates of false positives, moderate inter-reader reproducibility and radiology training burden, limiting its broad use outside expert centres [7–9].

Ultrasound modalities as well as their combination in a multiparametric approach are gaining increasing interest [10, 11]. Although conventional greyscale (GS) TRUS as imaging modality has a limited role in PCa diagnosis with sensitivity and positive predictive value (PPV) generally reported to be around 11–35% and 27–57%, respectively, ultrasound-based imaging offers many advantages [12, 13]. Ultrasound imaging is widely available, portable, less expensive in machine purchase and usage than MRI with the additional possibility of real-time imaging and biopsy needle monitoring. These advantages have motivated towards the development of various new ultrasound modalities striving to increase PCa detection including contrast-enhanced ultrasound (CEUS), computerized TRUS and (shear wave) elastography. Particularly, CEUS with quantitative parametric imaging and shear wave elastography (SWE) have produced encouraging results in recent studies [14, 15].

In CEUS, a suspension of gas-filled microbubbles, i.e. ultrasound contrast agents (UCAs) is used for visualization of microvascular flow patterns. Contrast-specific imaging is achieved by differentiating the non-linear scattering produced by the microbubbles from the linear tissue reflections. In PCa, abnormal blood flow patterns can be observed with CEUS and adding CEUS-targeted cores to the systematic biopsy procedure resulted in improved per-patient PCa detection rates [16, 17]. However, angiogenic microvascular cancer patterns can be ambiguous as higher blood flow by shunt formation and a higher microvascular density are counteracted by an increase in interstitial pressure and tortuosity [18]. To overcome this ambiguous effect of angiogenesis on blood flow limiting visual interpretation and perfusion-based quantification of CEUS, dispersion quantification techniques have been developed for a more detailed assessment of the UCA kinetics in the prostate. Several promising dispersion parameters have been extracted from recorded time-intensity-curves (TICs) and converted into parametric maps of the prostate with encouraging results in clinical prediction of PCa presence [14, 19].

SWE estimates tissue elasticity and can discriminate PCa, as malignancy typically shows increased stiffness, because of higher cellular density and collagen depositions [20]. In an SWE examination, an acoustic radiation force push pulse, induces a shear wave whose propagation is captured with an ultrafast ultrasonic imaging protocol. The speed of the shear wave is linked to the stiffness properties of the medium through which it propagates. SWE provides a dynamic quantitative map of soft-tissue elastic properties in near real time and is parametrically presented as a colour-coded overlay on the greyscale images [12, 21]. In two prostate biopsy studies, suspicious findings on SWE were at high risk of harboring clinically significant PCa while SWE targeted biopsy demonstrated equal per-core detection rates compared to systematic biopsy [22, 23]. Moreover, one study demonstrated that SWE allowed the identification of resection pathology proven PCa foci based on SWE density thresholds, potentially allowing for reader independent localization of prostate cancer foci [15].

Combining CEUS and SWE in a multiparametric ultrasound (mpUS) approach, in a similar fashion as mpMRI, could potentially reduce the risk of missing tumours that are not visible to one of the modalities and discriminate benign prostatic diseases like prostatitis that sometimes mimic malignant characteristics. Brock. et al. demonstrated in their mpUS study of 86 patients, with radical prostatectomy specimens as reference standard, that the addition of CEUS for lesions detected on strain elastography significantly decreased false-positive results (34.9% to 10.3%) and improved PPV from 65.1 to 89.7% [10]. With the known learning curve to perform strain elastography, the use of quantification software, inherent in SWE and as adjunct to CEUS, can not only improve diagnostic accuracy but also decrease user-dependency and training time while improving clinical applicability.

In this study protocol paper we will describe our present study evaluating the diagnostic accuracy of GS, SWE and CEUS with parametric mapping and its combination mpUS for the detection and localization of (clinically significant) PCa with radical prostatectomy specimens as reference standard. Additionally, this study will contribute to the development of a classifier algorithm, fully exploiting and integrating the complementary information of the different ultrasound modalities into a single parametric map.

## Methods/design

### Study objectives

#### Primary objectives

To determine the diagnostic performance i.e. sensitivity, specificity, PPV and negative predictive value (NPV) of GS, SWE, CEUS with quantitative mapping and their combination, mpUS, for the localization of clinically significant PCa foci.

Clinically significant PCa for the purpose of the primary objectives will be defined as the presence of a histopathologically confirmed Gleason ≥3 + 4 = 7 tumour focus with a tumour volume > 0.5 cm^3^.

#### Secondary objective(s)

To determine the diagnostic performance i.e. sensitivity, specificity, PPV and NPV of GS, SWE, CEUS with quantitative mapping and their combination mpUS for the detection and localization of PCa foci:

- for different thresholds of clinical significance; namely presence of a Gleason ≥4 + 3 = 7 tumour focus and presence of a Gleason ≥3 + 4 = 7 tumour focus, independent of volume, respectively
- in relation to the specific region of the prostate (peripheral zone versus transition zone)

To assess the technical feasibility, image quality and procedure related adverse events

To assess the interobserver agreement between US readers with difference levels of experience

To develop a classifier algorithm combining complementary information in the different ultrasound modalities into one single multiparametric map.

### Expected outcomes

It is expected that mpUS has the potential to improve PCa diagnosis and clinical decision making compared to currently applied diagnostic tests, as combining modalities has the potential to detect more tumours while being more specific as more different characteristics of suspicious lesions are evaluated. There is however limited data on the performance of combinations of ultrasound modalities [12]. Nelson et al. compared GS, Colour Doppler ultrasound and (strain) elastography targeted biopsies with sextant systematic biopsies as reference standard in 137 patients [24]. GS, Colour Doppler and elastography were positive in 16%, 29% and 25% of the 106 biopsy sites, respectively while the combination was positive in 46%, showing that the three modalities detect different tumours. Previously mentioned, Brock et al. demonstrated in their study that the addition of CEUS for lesions detected on real-time elastography decreased false-positive results and improved PPV [10]. None of these studies have included the quantitative techniques of our current study. Recently, Wildeboer et al. demonstrated in their study with 45 CEUS recordings, in 19 patients referred for radical prostatectomy, that the combination of CEUS parameters extracted from TICs performed better in detecting PCa than a single CEUS parameter with an accuracy of 81% for the combination compared to 73% for the best performing single parameter. Moreover the NPV increased to 83% from 70% [19]. Based on the available data, we expect that our mpUS will demonstrate higher diagnostic performance than the ultrasound techniques as stand alone.

### Study design

**Fig. 1:**
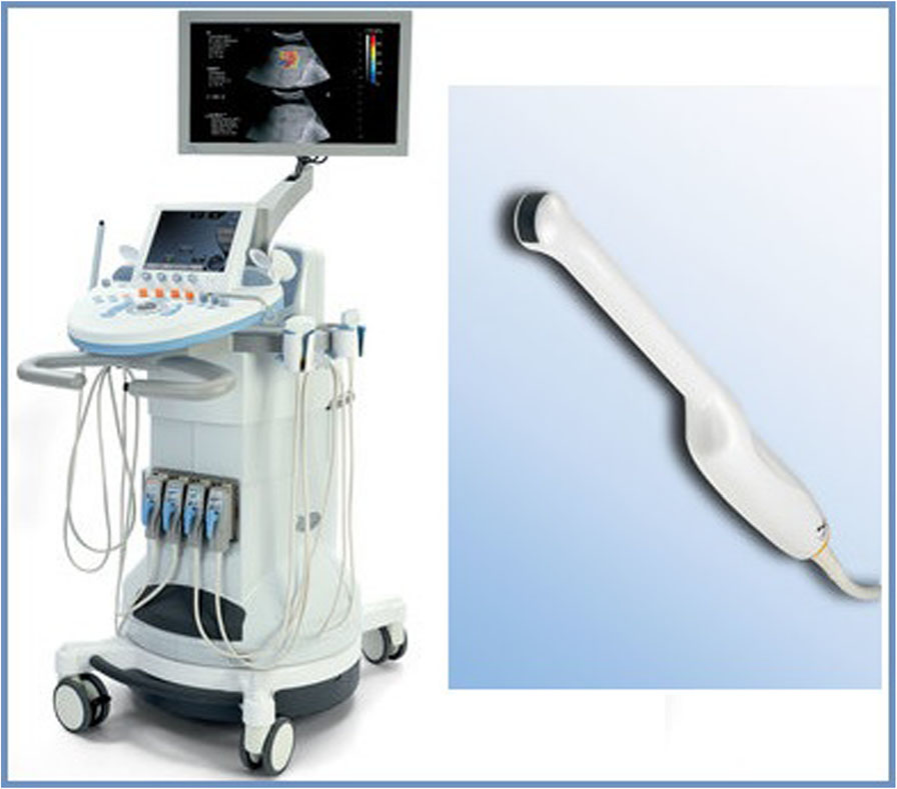
The ultrasound system and endorectal probe. Legend: Ultrasound scanner (Aixplorer®, Supersonic Imagine, Aix-en-Provence, France) and endorectal probe (SuperEndocavity™ SE12–3 with number of elements: 192 and bandwith: 3–12 MHz, Supersonic Imagine, Aix-en-Provence, France) used for the purpose of this study

This study is a prospective, single center, single group, in-vivo study in humans in which we will perform ultrasound imaging in patients with biopsy-proven PCa scheduled for radical prostatectomy. These patients will undergo ultrasound imaging using a clinical ultrasound scanner (Aixplorer®, Supersonic Imagine, Aix-en-Provence, France) with an endfire endorectal probe (SuperEndocavity™ SE12–3, Supersonic Imagine, Aix-en-Provence, France). The ultrasound scanner and probe are illustrated in Fig. 1. CEUS imaging requires the administration of a UCA bolus. SonoVue® (Bracco, Geneva, Switzerland), a well-tolerated and commonly used UCA, will be used through an intravenous cannula for the purpose of this study. After written informed consent, patients will undergo mpUS imaging the day prior to surgery. The prostate is examined in 3 planes (base, mid and apex of the prostate) using the 3 principal scanning modalities (GS, SWE and CEUS) sequentially. Usage of Colour Doppler and Power Doppler are left to the discretion of the ultrasound performer to avoid excessive scanning times. All scans will be recorded and exported as DICOM files with quantitative analysis of the CEUS recordings carried out remotely after the scan. At a later stage, recorded images will be evaluated independently by blinded readers, scoring the four quadrants of each imaging plane for the likelihood of clinically significant PCa based on a 1 to 5 Likert-scale for the different ultrasound modalities alone and for mpUS. Following resection, histopathologic analysis is performed according to institution standards with PCa tumour foci identified, graded and attributed to the imaging-derived quadrants in each prostate plane for all the prostatectomy specimens. Accurate registration of imaging and histopathology is reached using a standardized histopathology correlation protocol consisting of three-dimensional (3D) histopathological and imaging modelling, a registration procedure and a correlation step [25, 26]. This explorative study is in agreement with the IDEAL stage 2b recommendations [27].

### Population

#### Study population

The study population consists of the men with biopsy-proven prostate cancer that are scheduled for a radical prostatectomy. All patients will be recruited in the Martini Clinic*,* Prostate Cancer Center (Hamburg, Germany) and all study procedures will be performed in this institution. Patients will be informed about the study in oral and written form. Patient inclusion is confirmed by signing written informed consent. A total of 50 consecutive patients will be included in the study.

#### Inclusion and exclusion criteria

All inclusion and exclusion criteria are presented in Table 1. Exclusion criteria are based on contra-indications for ultrasound contrast agent usage and selected to allow a complete and reliable histopathological specimen analysis (no previous PCa therapy or hormonal therapy) and to maintain SWE image quality (upper prostate volume threshold of 80 mL). To better reflect the clinical practice where mpUS will be applied in the future if proven valuable, high risk patients with highly elevated PSA levels above 20 ng/mL and/or a clinical T3 digital rectal examination, will be excluded, as diagnostic imaging is less relevant in these patients.

**Table 1:** Inclusion and exclusion criteria

Inclusion Criteria	
1. Patients ≥18 years old	
2. Biopsy proven prostate cancer	
3. Treatment by radical prostatectomy (open or robot-assisted)	
4. Signed informed consent	
Exclusion Criteria	
1. PSA > 20 ng/mL and or clinical T3 rectal examination	
2. Prostate volume above 80 mL measured on TRUS	
3. Radiation therapy, focal therapy and/or chemotherapy for prostate cancer	
4. Inability to undergo TRUS	
5. Any form of hormonal therapy or androgen deprivation therapy within 6 months prior to procedure	
6. Any contraindication for the ultrasound contrast agent including cardiac right to left shunt, pulmonary hypertension, uncontrolled hypertension, instable coronary disease	
7. Has any medical condition or circumstance which would significantly decrease the chances of obtaining reliable data, achieving study objectives, or completing the study	

### Study procedures

#### Multiparametric ultrasound

After placement of an intravenous access, transrectal ultrasound imaging will be performed in the left-lateral decubitus position by one ultrasound performer. A total scanning time of 30 min is anticipated.

### Greyscale

After standard prostate volumetry and evaluation of the prostate capsule and seminal vesicles, transversal and sagittal sweeps of the entire prostate are slowly captured using GS ultrasound. Abnormal echogenicity patterns (calcifications, cysts and hypo-echoic lesions) are documented while the operator visually determines and stores pictures of the base, mid and apical transverse plane of interest taking into account the anatomical shape of the prostate. If areas of the prostate are considered more suspicious outside the anatomically chosen imaging planes, these are also brought into view and stored.

### Shear wave elastography

**Fig. 2:**
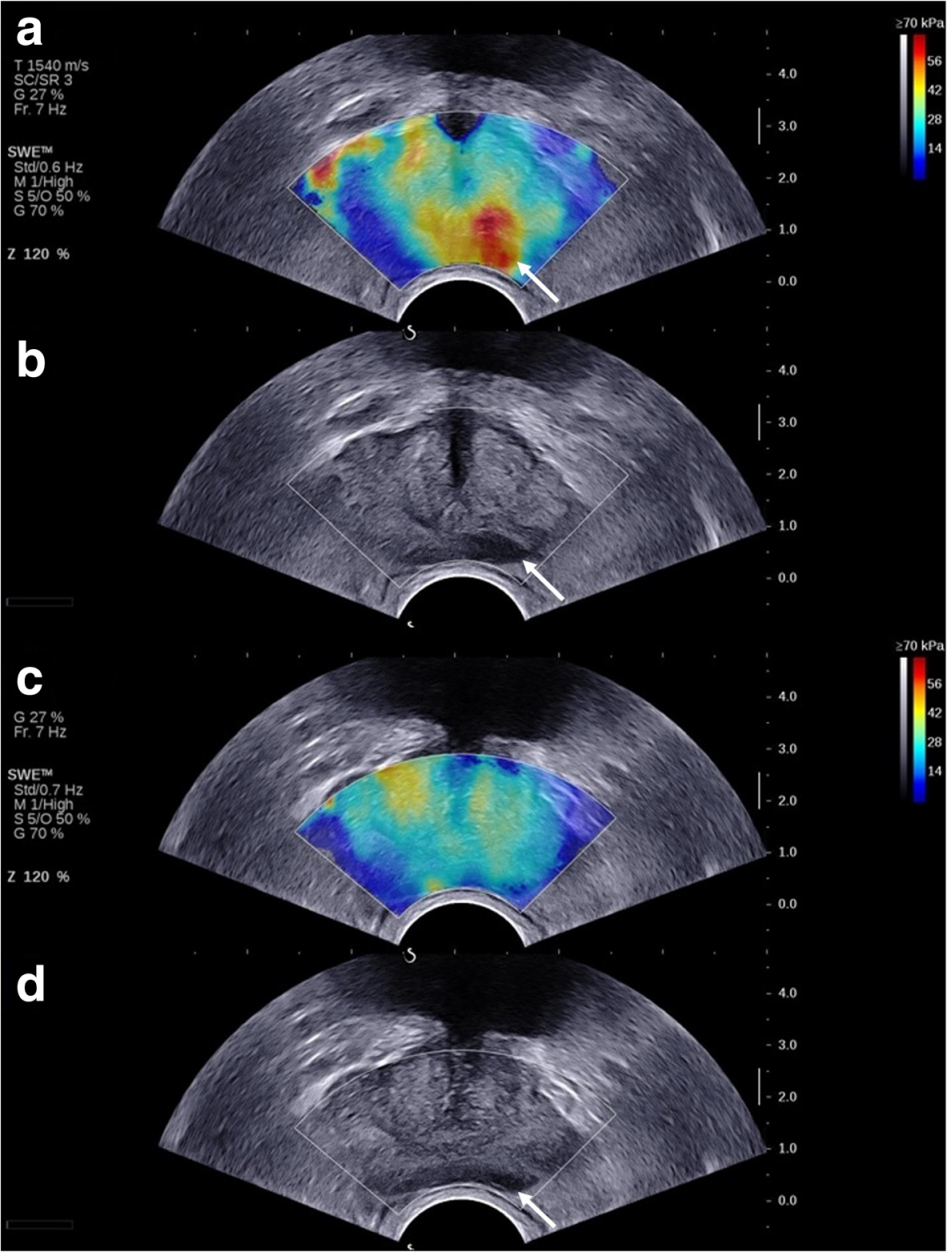
Shearwave elastography imaging of the prostate. Legend: An area with decreased tissue elasticity is visible in the left side of the prostate in the mid plane on SWE (white arrow) (**a**). This area is also visible as hypo-echogenious lesion on the corresponding greyscale image (white arrow) (**b**). A normal SWE pattern is visible in the base plane with the peripheral zone homogeneous coded in blue and the transition zone slightly heterogeneous in yellow (**c**). There is still some hypoechogenicity visible on the corresponding greyscale image (white arrow) (**d**). Radical prostatectomy revealed a Gleason 3 + 4 = 7 PCa with its primary focus in the left mid and apex of the prostate while the left base of the prostate was free of PCa tumour

Before SWE imaging, SWE specific settings (maximized penetration and appropriate elasticity scale) are checked and if necessary optimized while SWE examinations will be performed with minimal preload (pre-compressions). Each pre-defined transverse plane will be scanned with the SWE box in unilateral (left/right only) and bilateral (entire plane; maximum prostate plane coverage) fashion. For each scan, the transducer is maintained in a steady position for 5 s to make sure the signal is stable. Pictures and cine loops are stored for determination of elasticity values at a later stage. If areas of the prostate are considered more suspicious on SWE outside the predetermined imaging planes, these are also brought into view. An example of SWE is provided in Fig. 2.

### Contrast-enhanced ultrasound and quantification

CEUS settings (dynamic range, focus zone and mechanical index) are checked and optimized per patient. A total of 3 CEUS recordings will be made: One for each of the pre-defined planes. Each of the 2-min recordings will be started following the administration of a 2.4-mL bolus of UCA. After each recording a pause of 3 min is observed to allow the inflow of the next UCA bolus after sufficient UCA breakdown. A 4th bolus can be used if imaging quality due to e.g. patient movement is determined to be insufficient for quantitative analysis or for an area outside the imaging planes that is considered more suspicious on greyscale and/or SWE.

**Fig. 3:**
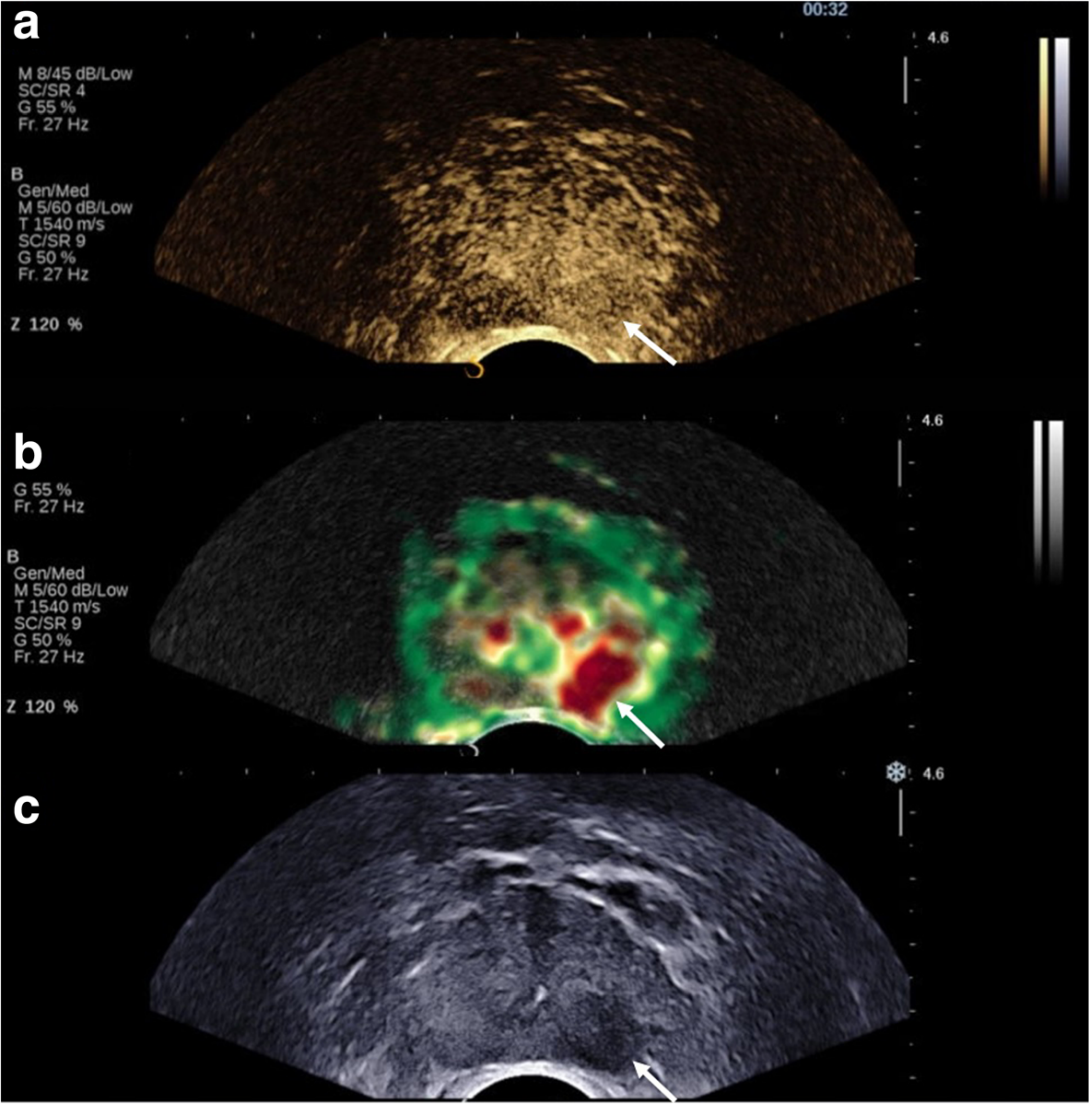
Contrast-enhanced ultrasound and contrast dispersion ultrasound imaging of the prostate. Legend: An area of early contrast enhancement is visible in the left peripheral zone of the prostate in the apical plane (white arrow) (**a**). Quantitative analysis with the Péclet CUDI parameter demonstrates a suspicious red lesion on the parametric image (white arrow) (**b**). The suspicious area is also visible as hypo-echogenious lesion on the corresponding greyscale image (white arrow) (**c**). Radical prostatectomy revealed a pT3a, Gleason 3 + 4 = 7 with tertiary pattern 5 PCa on the left apical side of the prostate

CEUS recordings will be stored and transferred for quantitative analysis. In this study, we will use the Contrast Ultrasound Dispersion Imaging (CUDI) analysis of the Eindhoven University of Technology with computer-aided quantification and parametric mapping [28, 29]. In short, this method quantifies the dispersive effects in the contrast concentration kinetics on a pixel basis by spatiotemporal analysis of the UCA in- and outflow during the CEUS recording. Several dispersion parameters have been derived that show promising results in prediction of PCa presence using radical prostatectomy specimens as the reference standard with a receiver-operating-characteristic (ROC) area under the curve (AUC) ranging from 0.84–0.89 [28, 30–32]. The resulting colour-coded parametric maps can be used to assess PCa presence. An example of CEUS and CUDI is provided in Fig. 3.

### Radical prostatectomy and histopathology

The radical prostatectomy (open or robot-assisted laparoscopic) will be performed in accordance to institution standards. In the majority of patients (> 90%) an intraoperative neurovascular structure-adjacent frozen section examination technique will therefore be performed. In this procedure, frozen sections are taken from one or both lateral side(s) of the prostate to enable the sparing of nerves while decreasing positive surgical margins [33]. These frozen sections are processed separately from the resected specimen in the pathology lab. Following the radical prostatectomy, the whole specimen and frozen sections will be macroscopically photographed. The resection specimen is fixated and dissected in 4-mm thick transversal slices and quadrant sections with the location and orientation of all coupes recorded. Pathologic analysis will be performed by dedicated genitourinary pathologists, unaware of imaging results, who will evaluate the entire specimen for presence of tumour (marking each lesion’s Gleason score), extracapsular invasion and seminal vesicle invasion. Individual foci of tumour will be outlined.

### Histopathologic correlation of imaging

All US imaging, each sequence separate and combined, will be evaluated by US readers in blinded fashion. The four quadrants (left and right peripheral zone and left and right transition zone, respectively) of each imaging plane (base, mid and apex) will be evaluated for the likelihood of clinically significant PCa based on a five-point Likert Scale (1: highly unlikely; 2: unlikely; 3: equivocal; 4: likely; and 5: highly likely), resulting in a total of 12 regions of interest (ROIs) per prostate. Examiners are blinded for clinical and pathological data but aware that patients are scheduled for radical prostatectomy.

**Fig. 4:**
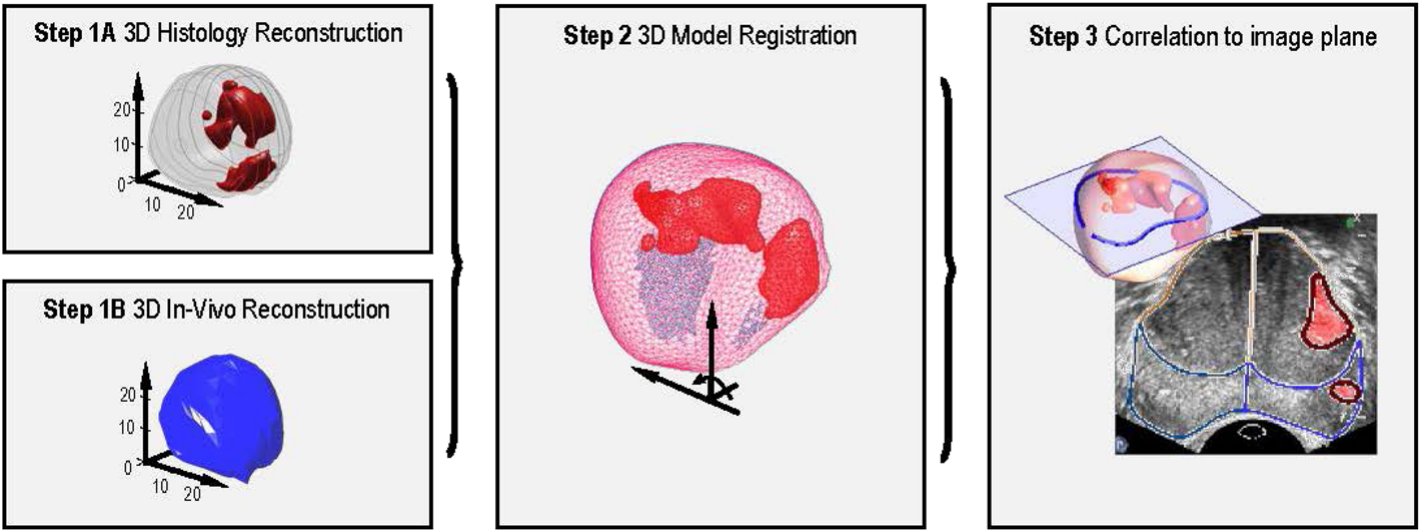
Schematic overview of a full registration framework for the correlation of the ultrasound image with histopathology. Legend: A 3D reconstruction of the ex-vivo radical prostatectomy specimen and in-vivo gland (Step 1A and 1B); registration between the in-vivo and ex-vivo model (Step 2); Correlation of the pathology data and ultrasound image (Step 3); Pixel-wise superposition of the histopathological data onto the ultrasound image

Matching of US imaging with histopathology is a challenge; not only do the deformations of the prostate after resection have to be taken into account, but also the mismatch in orientation between imaging planes and pathology slices and deformation due to transrectal probe usage. To provide for an accurate histopathologic correlation a three-step process combing reconstruction, registration and correlation is used in line with our previous published work [25, 26, 34]. First, a 3D histopathological reconstruction with adequate interpolation of tumour delineations into tumour volumes is performed based on the pathology slices. Hereafter, a 3D US model of the in-vivo prostate is reconstructed from the 2D greyscale sweeps [25, 26]. Thirdly, a 3D, surface-based elastic registration method is used to fuse the in-vivo 3D US model with the 3D histopathological model. This method avoids the need of landmarks or a high level of detail, often lacking in greyscale US, while no manual intervention is required during the registration [26]. Lastly, the registered 3D models are correlated to the actual images with superposition of histopathology onto the ultrasound imaging with its 12 ROIs per prostate. Figure 4 provides a schematic overview of a full registration procedure.

### Statistical analysis and sample size

Demographic and disease specific characteristics of the study population will be descriptively reported. For localization of PCa, a logistic generalized estimating equation (GEE) model, accounting for the fact that 12 ROIs will be analysed in the same patient, will be used to estimate the sensitivity, specificity, PPV and NPV for the three different US modalities and any combination of those, both for different Likert scale thresholds (Likert ≥3 and Likert ≥4) as for the predefined criteria of clinically significant PCa. In principle, the model will contain US modality, reader and their interaction. Sensitivity is defined as the probability of correctly identifying a tumour focus in a given ROI. Specificity is defined as the probability of correctly identifying ROIs negative for tumour. The effect of histopathological variables (Gleason score, lesion size and pT-stage) will be tested for the sensitivity of each US modality. For detection, readers with Likert scores ≥4 for any clinically significant PCa-containing ROI are considered to have detected PCa in that particular patient. The interobserver agreement will be evaluated using the intraclass correlation coefficient.

No formal sample size calculation was performed. In line with the IDEAL recommendations for explorative studies and published (mpMRI) studies with a similar design a total of 50 patients will be included in the study [35–37].

### Quality and patient safety

Quality of data and patient safety will be continuously monitored by the investigators. Periodical reporting of study progression and patient safety will be performed to the reviewing Institutional Review Board (IRB). The investigator will inform the subjects and the reviewing IRB if anything occurs of which the disadvantages of participation may be significantly greater than was foreseen in the research proposal. The investigators will notify the IRB without undue delay of a temporary halt including reason for such an action. The investigators will take care that all subjects are kept informed.

### Risks and benefits

TRUS imaging of the prostate is safe and well tolerated. There is a small anticipated risk in this study because of the UCA usage. After its use in thousands of patients, adverse events related to UCAs appear to be mild, rare and transient [38–40]. Sensations of warmth, facial or general flushes and itching (at injection site) are the most frequently reported minor side effects. Serious adverse reactions, which consists of hypersensitivity allergic reactions are rare (< 0.01%), but comprehensive rescue measures are prepared and available for all the patients in the study. There is no direct benefit for patients included in this study. Results of this study, however, can be important for future patients in the diagnostic work-up for PCa. Patients will be informed of the risks and absence of benefit, and both will be described in the study information

## Discussion

New ultrasound modalities with quantitative techniques, such as SWE and CEUS with parametric mapping, are gaining interest. The exploration of these techniques in a multiparametric fashion is essential for the development of an ultrasound-based imaging approach with the potential of real-time PCa imaging and targeted biopsy. This study, including a ground truth reference standard, will give insight into different US features of PCa and into its combined diagnostic value. Furthermore this study will provide information on the question whether mpUS could potentially be used as a triage test to exclude significant PCa or should be used to target specific regions suspicious for significant PCa or both. With accurate registration and fusion gaining attention for reliable image-targeted biopsies and (focal) treatment, we believe that our study also contributes to the introduction of suitable registration/fusion options.

Its design with radical prostatectomy specimens as reference standard, however also comes with some disadvantages. First, the population is different from the primary diagnostic setting since men must have PCa and choose to have surgery (spectrum bias). Second, the reader examining the images is aware that there must be PCa, potentially biasing readers towards higher sensitivity readings (observer bias). However, studies using a more appropriate population with prostate biopsy specimens as reference standard, fail to detect all clinically significant lesions found after radical prostatectomy, even in a template-guided transperineal saturation setting [1, 41]. Despite this important limitation of prostate biopsy as reference standard, a biopsy study can be foreseen as next step in the clinical assessment of mpUS imaging for PCa diagnosis. After all, the clinical utility of a targeted biopsy approach for mpUS suspicious lesions cannot be accurately assessed in this study as a targeted biopsy procedure is not only dependent on the scoring of lesions on mpUS but also dependent on other factors such as targeting accuracy, biopsy operator experience and patient movement.

Another limitation of this study is that the prostate is evaluated in 2D US imaging planes. There is a risk of missing tumours outside the predefined imaging planes while the UCA transport kinetics have to be modelled with strong assumptions in its directionality [31]. Although a 3D approach can overcome these limitations and reduce the number of UCA bolus injections, no clinical US device is currently available with both 3D SWE and 3D CEUS imaging.

We have chosen for a stringent 12 region based template per prostate for our analysis. Although, in comparison with the prostate imaging reporting and data system version 2 (PI-RADSv2), this is a limited number of regions, we assume that this approach is the best approximation for US and pathology matching as more ROIs per template would increase the risk of registration errors. Besides the well-known errors in the registration procedure of ultrasound imaging and pathology caused by gland deformation, fixation-related shrinkage and a mismatch in US imaging and pathology orientation, the intraoperative frozen section examination have to be taken into account in our study [42, 43]. To assess the influence of the registration between imaging and histopathology on the final results, separate analyses including and excluding ROIs with small tumors (with respect to the ROIs) and inconsistencies across multiple ROIs are foreseen. Besides, studies with PI-RADSv2 or more ROIs per template often also simplify their template for analysis or use more tolerant approaches for misalignment with inclusion of neighboring regions [44–46].

Lastly, further research regarding improvements to the standardization and reproducibility of these ultrasound modalities as stand-alone tools and in a multiparametric fashion is still required. Various ultrasound manufacturers have introduced SWE into their ultrasound scanners and computer-aided quantification and parametric mapping of CEUS recording with CUDI is not limited to a specific ultrasound scanner. Therefore, there is an increasing need to define quality criteria for these new techniques, provided that results of our study are positive, in order to improve clinical application and generalizability of these techniques in other centres with their own local expertise and resources [47, 48].

Despite these limitations, we expect that the results of this study will contribute to the assessment of the role of mpUS imaging for the diagnosis of PCa in clinical practice. In light of current limitations of prostate biopsy and mpMRI, mpUS holds the potential for an accessible imaging-based PCa approach.

## Abbreviations

(PI-RADSv2): Prostate imaging reporting and data system version 2
AUC: Area under the curve
CEUS: Contrast - enhanced ultrasound
CUDI: Contrast ultrasound dispersion imaging
DRE: Digital rectal examination
EAU: European Association of Urology
GS: Gleason score
IRB: Institutional Review Board
mpMRI: Multiparametric magnetic resonance imaging
mpUS: Multiparametric ultrasound
NPV: Negative predictive value
PCa: Prostate cancer
PPV: Positive predictive value
PSA: Prostate specific antigen
SWE: Shear wave elastography
TIC: Time-intensity-curve
TRUS: Transrectal ultrasound
UCA: Ultrasound contrast agent
